# Effect of botanical drugs in improving symptoms of hypertensive nephropathy: Analysis of real-world data, retrospective cohort, network, and experimental assessment

**DOI:** 10.3389/fphar.2023.1126972

**Published:** 2023-04-04

**Authors:** Jia-Ming Huan, Xi-Ting Ma, Si-Yi Li, Dong-Qing Hu, Hao-Yu Chen, Yi-Min Wang, Xiao-Yi Su, Wen-Ge Su, Yi-Fei Wang

**Affiliations:** ^1^ School of Traditional Chinese Medicine, Shandong University of Traditional Chinese Medicine, Jinan, China; ^2^ School of Chinese Medicine, Hong Kong Baptist University, Kowloon, Hong Kong SAR,China; ^3^ Medical Services Section, Affiliated Hospital of Shandong University of Traditional Chinese Medicine, Jinan, China; ^4^ Department of Cardiovascular, Affiliated Hospital of Shandong University of Traditional Chinese Medicine, Jinan, China

**Keywords:** hypertensive nephropathy, real-world data, machine learning, NF-κB signal pathway, clinical decision support

## Abstract

**Background/aim:** Hypertensive nephropathy (HN) is a common complication of hypertension. Traditional Chinese medicine has long been used in the clinical treatment of Hypertensive nephropathy. However, botanical drug prescriptions have not been summarized. The purpose of this study is to develop a prescription for improving hypertensive nephropathy, explore the evidence related to clinical application of the prescription, and verify its molecular mechanism of action.

**Methods:** In this study, based on the electronic medical record data on Hypertensive nephropathy, the core botanical drugs and patients’ symptoms were mined using the hierarchical network extraction and fast unfolding algorithm, and the protein interaction network between botanical drugs and Hypertensive nephropathy was established. The K-nearest neighbors (KNN) model was used to analyze the clinical and biological characteristics of botanical drug compounds to determine the effective compounds. Hierarchical clustering was used to screen for effective botanical drugs. The clinical efficacy of botanical drugs was verified by a retrospective cohort. Animal experiments were performed at the target and pathway levels to analyze the mechanism.

**Results:** A total of 14 botanical drugs and five symptom communities were obtained from real-world clinical data. In total, 76 effective compounds were obtained using the K-nearest neighbors model, and seven botanical drugs were identified as Gao Shen Formula by hierarchical clustering. Compared with the classical model, the Area under the curve (AUC) value of the K-nearest neighbors model was the best; retrospective cohort verification showed that Gao Shen Formula reduced serum creatinine levels and Chronic kidney disease (CKD) stage [OR = 2.561, 95% CI (1.025–6.406), *p* < 0.05]. With respect to target and pathway enrichment, Gao Shen Formula acts on inflammatory factors such as TNF-α, IL-1β, and IL-6 and regulates the NF-κB signaling pathway and downstream glucose and lipid metabolic pathways.

**Conclusion:** In the retrospective cohort, we observed that the clinical application of Gao Shen Formula alleviates the decrease in renal function in patients with hypertensive nephropathy. It is speculated that Gao Shen Formula acts by reducing inflammatory reactions, inhibiting renal damage caused by excessive activation of the renin-angiotensin-aldosterone system, and regulating energy metabolism.

## 1 Introduction

Hypertensive nephropathy (HN) is one of the common complications of hypertension and can significantly increase the prevalence of chronic kidney disease (CKD) (Ene-Iordache et al., 2016; Williams et al., 2018). According to the current guidelines (Hart and Bakris, 2010; Ene-Iordache et al., 2016; Carey et al., 2018; Williams et al., 2018), patients with HN should not only manage their blood pressure but should also consider renal protection to obtain long-term benefits. Glomerulosclerosis is the most common renal pathological change caused by hypertension. Long-term hypertension leads to renal arteriosclerosis, transparent changes in glomerular arterioles, increased renal vascular resistance, inflammatory reactions, and renal tubulointerstitial fibrosis (Seccia et al., 2017). Patients typically first present with microalbuminuria and glomerular ischemia aggravated to decompensation, followed by albuminuria, elevated serum creatinine levels, polyuria, increased nocturia, and other symptoms (Bakris, 2004; Ibsen et al., 2005; Hart and Bakris, 2010). Data from randomized controlled trials show that lowering blood pressure while inhibiting the renin-angiotensin-aldosterone system (RAAS) yields additional benefits for patients with urinary albumin/creatinine ratios greater than 33.9 mg/mmol (Bakris et al., 2010; Schmieder et al., 2011).

Botanical drugs have advantages in improving HN symptoms. In the theory of traditional Chinese medicine (TCM), the use of botanical drugs emphasizes the symptom characteristics of the population. According to existing research (Owoicho Orgah et al., 2018; Wu et al., 2018; Yan et al., 2018; Li et al., 2020), compared with the use of antihypertensive drugs alone, the combined use of chemical drugs and traditional Chinese medicine prescriptions can more effectively reduce the uncomfortable symptoms of patients, such as vertigo, poor appetite, fatigue, drowsiness, frequent nocturnal urination, and lower limb edema. However, most of these conclusions are based on observational case‒control studies, and evidence from well-designed randomized controlled trials is lacking.

Botanical drugs are rich in compounds that act on multiple targets of the disease and improve the imbalance of the disease biological networks (Zhao et al., 2010). HN involves a number of complex pathological changes and is associated with a large number of combinations of symptoms and signs. Using a complex network based on real-world data (RWD), it is possible to collect relevant information about diseases from electronic medical records (EMRs) and biological databases and to analyze the prescription network of botanical drugs in RWD, thereby decreasing the difficulties caused by the diversity of the clinical manifestations of diseases (Wang et al., 2019; Yang et al., 2021). Existing studies (Wang et al., 2021) show that small molecules present in botanical drugs can not only act on disease-related genes but also play a role in interfering with the disease process through biological networks. TCM prescriptions need to aim at a variety of symptoms and signs. Using network pharmacology combined with a machine learning algorithm, we can further explore the potential relationship between botanical drugs and diseases, analyze network integration of botanical drugs and disease phenotypes at the level of protein interactions to show the effect of botanical drugs on disease-related molecular networks, and explain the relevant mechanisms of action (Liu, 2013; Yang et al., 2020).

**FIGURE 1:**
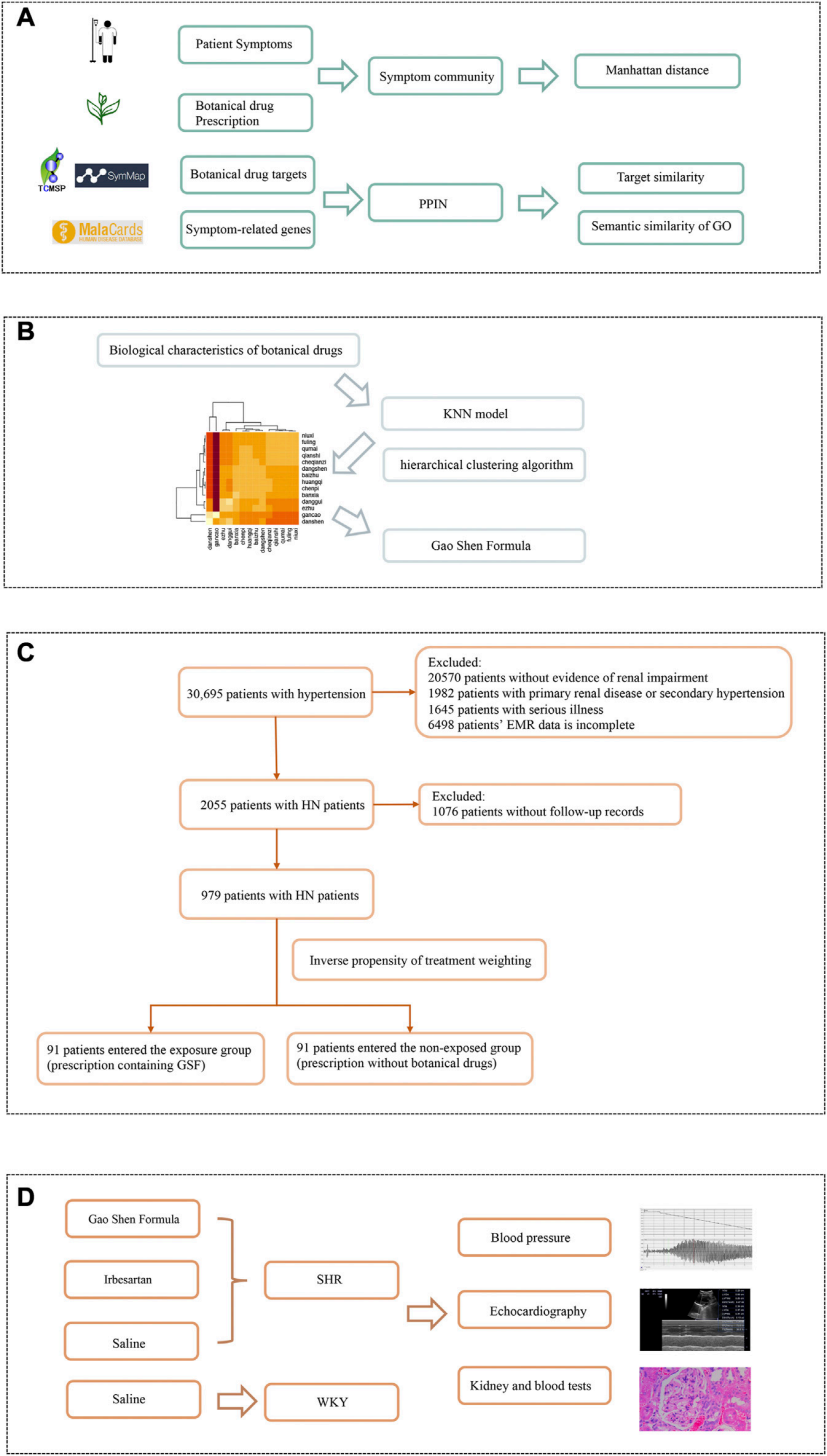
Workflow of botanical drugs screening and efficacy verification. **(A)** Establishing symptom–botanical drug relationships. Based on the symptom network and protein–protein interaction network, we quantified the biological characteristics of botanical drugs. **(B)** Analysis of botanical drug characteristics. The KNN model was used to comprehensively analyze the biological characteristics of botanical drugs to form a correlation heatmap of the interaction between botanical drugs. After hierarchical clustering algorithm screening, the composition of the Gao Shen Formula is determined. **(C)** Regression cohort validation. We screened patients based on the inclusion and exclusion criteria. After propensity score matching, the exposed group and the non-exposed group each included 91 patients. **(D)** Experimental model verification. We used Gao Shen Formula, irbesartan, and saline to receive SHR or WKY for 8 weeks, and observed the differences of blood pressure, echocardiography, kidney, and blood indexes in rats.

This study was based on data derived from the EMRs of patients with HN. The hierarchical network extraction algorithm and the fast unfolding algorithm were used to summarize the network information on patients' prescriptions and disease symptoms. A machine learning model was used to comprehensively analyze the clinical information and biological characteristics and obtain the core prescription Gao Shen Formula (GSF). Spontaneously hypertensive rats were used to establish a model for the verification of the mechanism of action of GSF (Figure 1).

## 2 Materials and methods

### 2.1 Data preparation

A total of 30,695 electronic medical records of patients diagnosed with hypertension were collected from the Affiliated Hospital of Shandong University of Traditional Chinese Medicine from 1 July 2014 to 31 May 2017. A total of 2,055 patients with HN were extracted. The diagnosis, demographic information (such as age and sex), chief complaint, medication prescriptions, and results of auxiliary examinations were collected from the patients’ EMRs. The patients’ symptoms were described using standard terminology, and the database was standardized with reference to the Medical Subject Headings (MeSH) and the *Chinese Pharmacopoeia*.

### 2.2 Botanical drug combination for the treatment of HN

In this study, prescriptions were extracted from the EMRs of HN patients. Setting botanical drugs as the node and the frequency of occurrence of two botanical drugs in different prescriptions as weights, a weighted prescription network was established. The hierarchical network extraction algorithm (Zhou et al., 2010) was used to find the core botanical drugs in the network. At the same time, relative risk (RR) was used to identify the botanical drugs that were specifically used to treat patients with HN. The prescription used to treat HN was set as the exposure group, the remaining cases comprised the non-exposure group, and the single botanical drug used in the exposure group was the final event. The botanical drugs extracted by the hierarchical network and RR were combined and regarded as the botanical drug combination for treating HN.

### 2.3 Module division of HN symptoms

The fast unfolding algorithm (Blondel et al., 2008) has been widely used in data mining. In this study, a symptom network of patients with HN was extracted and established from EMRs. The fast unfolding algorithm was used to find various combinations of symptoms. *Gephi* (0.9.4) was used to implement the fast unfolding algorithm.

### 2.4 Interaction network of botanical drugs and symptoms

#### 2.4.1 Compounds and targets of botanical drugs

The compounds and targets of core botanical drugs were collected from public databases, including the Traditional Chinese Medicine Systems Pharmacology Database and Analysis Platform (TCMSP) (Ru et al., 2014), SymMap (Wu et al., 2019), the Encyclopedia of Traditional Chinese Medicine (ETCM) (Xu et al., 2019), PubMed, and CNKI databases. The PubChem and UniProt databases were used to unify the information on compounds and targets.

#### 2.4.2 HN-related genes

HN-related genes were collected from the GEO database. The data were analyzed online using the GEO2R tool and supplemented using data from several mature databases, including Online Mendelian Inheritance in Man (OMIM) (Amberger et al., 2015), DisGeNET (Amberger et al., 2015), and Malacards (Rappaport et al., 2017). Symptom-related genes were obtained in the SymMap database.

#### 2.4.3 Protein–protein interaction network

STRING (Szklarczyk et al., 2021) is a network-weighted protein‒protein interaction database that integrates a variety of data sources, including experimental data, algorithm prediction, and literature mining. The regulatory targets of botanical drugs and genes related to HN were input into the *Homo sapiens* database to establish the protein‒protein interaction network (PPIN). In this study, shortest path analysis was used to identify the botanical drugs that are closely related to HN in the PPIN. The Dijkstra algorithm is a classical network node shortest-distance algorithm that is used to calculate the shortest distance between nodes in the PPIN. Nodes with a shortest distance of less than three were defined as strongly connected nodes (Yang et al., 2020).

### 2.5 Biological characteristics of botanical drugs

Information on the biological characteristics of the botanical drug compounds was used to analyze their effectiveness. The degree of regulation of HN was evaluated using information such as bioavailability and target importance.

#### 2.5.1 Evaluation of symptom community

To evaluate the regulation of each HN symptom by different compounds, this study summarized the number of genes regulated by different compounds in different symptom communities and established a frequency matrix. The Manhattan distance (Man) implemented in the *proxy* package (version 0.4-26) in R 3.6.3 was used to evaluate the compactness of compounds in each module in the matrix.

#### 2.5.2 Oral bioavailability

Oral availability reflects the extent to which compounds are absorbed by the gastrointestinal tract and metabolized by the liver and is used as an important indicator of a compound’s effectiveness (Xu et al., 2012). The OB values of compounds were collected from the TCMSP and SymMap databases.

#### 2.5.3 Target similarity

The Jaccard similarity coefficient (Jac) is used to measure the similarity between datasets. In this study, a Jac value was calculated for each compound target. The average Jac value was used to quantify the similarity of the botanical drug targets.

#### 2.5.4 Similarity of biological processes

The semantic similarity of Gene Ontology (GO) provides a method for calculating similarities among genes. In this study, GO biological process semantic similarity (GoSim) was used to measure the similarity between regulatory genes affected by compounds and biological processes related to HN. *BioConductor*, implemented in the *GOSemSim* package (version 2.12.1) in R 3.6.3, was used to provide annotation data (Wang et al., 2007).

### 2.6 Model analysis of botanical drug characteristics

K-nearest neighbors (KNN) is a supervised machine learning algorithm that can generate a variety of feature information about compounds, match the input features with the expected output to create a learning function, and complete the classifier after cross-learning to adjust the parameters. The KNN model is sensitive to local information on interactions. KNN can be used to reflect the connection characteristics of input compounds and is more reliable for analysis of the targets of botanical drugs (Wang et al., 2019). In this study, the biological characteristics of the compounds were input into the KNN model and used to predict their relevance to HN.

To determine the screening criteria to be used in the model, the random walk with restart (RWR) algorithm was used to find the key compounds. The HN-related gene was set in the PPIN as the seed node, and the restart probability was 0.75 (Yang et al., 2020). The lower quartile of the correlation score calculated by RWR was set as the screening threshold for important compounds, and screening was implemented in the *pyrwr* package (version 1.0.0) in Python 3.7.5.

The bioinformatics characteristics of botanical drug compounds (including OB, Jac, GoSim, and Man) were used as the input information for the KNN model and implemented in the *kknn* package (version 1.3.1) in R 3.6.3. To avoid data waste, the training uses 10 cross-validations to complete the model performance evaluation (Wang et al., 2019).

To evaluate the performance of the KNN model, three other KNN control models were designed; they included input only OB (Comparison 2), input only Jac (Comparison 3), and input only gene regulatory information including GoSim and Man (Comparison 4).

Receiver operating characteristic (ROC) curves of the four models were drawn, and the area under the curve (AUC) values were calculated to determine the effectiveness of the original model. After determining the best model, the point closest to the upper left corner of the ROC curve was used as the threshold point. Machine learning models such as support vector machines (SVMs), gradient boosting decision trees (GBDTs), and Bayesian networks (BNs), which are widely used in botanical drug bioinformation network analysis (Wang et al., 2007; Blondel et al., 2008; Yu et al., 2010; Zhou et al., 2010; Xu et al., 2012; Liu, 2013; Ru et al., 2014; Sinwar and Kaushik, 2014; Amberger et al., 2015; Piñero et al., 2016; Rappaport et al., 2017; Wang et al., 2019; Wu et al., 2019; Xu et al., 2019; Yang et al., 2020; Szklarczyk et al., 2021; Wang et al., 2021; Huan et al., 2022), were compared with the KNN model to determine the effectiveness of each model.

### 2.7 Botanical drug screening

The KNN model can perform the screening of effective compounds. To effectively evaluate the effectiveness of botanical drugs, this study calculated the number of effective compounds contained in each botanical drug and used the hierarchical clustering algorithm (HCT) to classify and screen botanical drugs. In this study, Euclidean distance is used, and the clustering method of the Ward (1963) rule is used. Based on the results obtained in this way, the effective part of the core combination of botanical drugs was determined and used as an effective prescription, GSF, for the treatment of HN.

### 2.8 Pathway enrichment

In this study, the *org.Hs.e.g.db* package (version 3.10.0) in R 3.6.3 was used to annotate the regulatory genes affected by GSF, the *clusterProfiler* package (version 3.14.3) (Yu et al., 2012) in R 3.6.3 was used for Kyoto Encyclopedia of Genes and Genomes (KEGG) enrichment analysis, Q < 0.05 was used to define significant enrichment pathways, and GO enrichment analysis was performed using the same method. Metascape (Zhou et al., 2019) was used to summarize the genetic information for each symptom module. The molecular complex detection (MCODE) algorithm, implemented in the MCODE package (version 2.0.2) in Cytoscape 3.9.1, was used to analyze the interaction sets of the core targets of GSF in the PPIN, to analyze the degree of closeness of each HN symptom, and to identify the regulatory mechanism through which GSF acts.

### 2.9 Clinical efficacy of GSF

To evaluate the reliability of the KNN model and the HCT screening results, a retrospective cohort study was designed and used to evaluate the efficacy of GSF treatment based on real-world data. The retrospective cohort was established by analyzing the EMRs of 2,055 HN patients, screening both patients whose prescriptions included botanical drugs composed of GSF and patients who were not treated with botanical drugs, and evaluating changes in their clinical indices after six months of treatment. To ensure a balance among the selected patients, the tendency score was used to comprehensively quantify baseline factors such as age, sex, hospitalization time, and renal function, and the inverse probability processing weighted model was used. Inverse propensity of treatment weighting (IPTW) (Austin and Stuart, 2015), implemented in the *Matching* package (version 4.911) in R 3.6.3, was used to screen for matching patients, stabilize the tendency score between groups, and achieve a baseline balance among the patients.

The inclusion criteria were as follows: 1) a history of hypertension for more than five years; 2) persistent microalbuminuria or mild to moderate albuminuria during the course of the disease; 3) renal dysfunction caused by primary renal disease with hypertension and exposure to nephrotoxic substances, congenital or hereditary kidney disease, or other diseases; 4) a clear diagnosis in the medical record; and 5) the patient’s data in the medical record are complete.

The exclusion criteria were as follows: 1) secondary hypertension; 2) severe cardiovascular or cerebrovascular disease, malignant tumors, severe hematopoietic system, respiratory system, digestive system, or infectious disease; 3) incomplete auxiliary examination of renal function during hospitalization; and 4) hospitalization time less than 1 week.

The data of each eligible patient began with the electronic medical record recorded during hospitalization. The items collected include 1) the patient’s demographic characteristics (name, sex, and age); 2) clinical features including length of hospital stay, serum creatinine, uric acid, urinary protein, blood lipids, and other parameters; and 3) details of the patient’s treatment, including the composition of any traditional Chinese medicine prescriptions used. The estimated glomerular filtration rate (eGFR) was estimated using the CKD-EPI formula (Levey et al., 2009). The CKD staging of the patients was based on the Clinical Practice Guideline for the Evaluation and Management of Chronic Kidney Disease that appear in Kidney Disease: Improving Global Outcomes (KDIGO) (Kidney Disease: Improving Global Outcomes (KDIGO) CKD Work Group, 2012).

The sample size was estimated based on reports in the clinical literature and on the previous research performed by the research group. The effective rate of modern medicine for symptomatic treatment is approximately 71% and that of combined traditional Chinese medicine is approximately 88%. The formula used to calculate the sample size is as follows:

$$n=\frac{{\left[Z_{\alpha }\sqrt{2\bar{p}\left(1-\bar{p}\right)}+Z_{\beta }\sqrt{p_{1}\left(1-p_{1}\right)+p_{0}\left(1-p_{0}\right)}\right]}^{2}}{{\left(p_{1}-p_{0}\right)}^{2}},$$

where $p_{0}$ is the effective rate of the control group and is set to 71% and $p_{1}$ is the effective rate of the exposure group and is set to 88%. $\bar{p}$ is the average value of $p_{0}$ and $p_{1}$ . $Z_{\alpha }$ and $Z_{\beta }$ are the quantiles of the standard normal distributions of α and β , respectively. This study is a one-sided test, α=0.05 and β=0.1 , then $Z_{\alpha }$ = 1.65 and $Z_{\beta }$ = 1.28. The required sample size for each group was calculated to be 95.

The traditional Chinese medicine prescriptions taken by patients in the GSF group included the constituent drugs of GSF. During the 6-month study period, all patients had records of traditional Chinese medicine prescriptions during outpatient visits or hospitalization in the Affiliated Hospital of Shandong University of Traditional Chinese Medicine and blood or urine test results related to their treatment with traditional Chinese medicine. During this period, all patients took antihypertensive drugs and patients with a GFR greater than 60 $ml/\left(\min∙1.73\;m^{2}\right)$ were treated with angiotensin receptor blocker drugs, including irbesartan 75–150 mg/d. Patients with a GFR less than 60 $ml/\left(\min∙1.73\;m^{2}\right)$ were treated with calcium channel blockers drugs, including nifedipine control tablets 30–60 mg/d.

### 2.10 Experimental model verification of GSF

#### 2.10.1 Experimental design

Thirty 14-week-old male spontaneously hypertensive (SHR) rats with body weights of 280–300 g were purchased from SPF (Beijing) Biotechnology Co., Ltd., China [specific pathogen-free level, certificate No. SCXK (Beijing) 2019-0010]. Ten age-matched Wistar Kyoto (WKY) rats with body weights of 280–300 g were purchased from Beijing Vital River Laboratory Animal Technology Co., Ltd., China [specific pathogen-free level, certificate No. SCXK (Beijing) 2021-0006]. Chinese granule herbal extracts of GSF were purchased from Tiangjiang Pharmaceutical Co., Ltd, China. Irbesartan tablets [lot No. 0000009987] were purchased from Zhejiang Huahai Pharmaceutical Co., Ltd. China. The SHR and WKY rats were housed in standard plastic cages with a 12-h light/dark cycle at 23°C ± 1 °C and given free access to food and water. After a 10-week acclimation period, the 24-week-old SHR rats were randomly divided into three groups: 1) a GSF group in which each animal was treated with Chinese granule herbal extract at 4.5 g/kg body weight per day (i.g.); 2) a positive group in which each animal received irbesartan tablets 13.5 mg/kg body weight per day (i.g.); and 3) an SHR group in which each animal received 2 mL of saline per day (i.g.). The WKY group consisted of 10 rats, each of which received 2 mL of saline per day (i.g.). The intervention lasted for 8 weeks. The study was approved by the Animal Ethics Committee of the Affiliated Hospital of Shandong University of Traditional Chinese Medicine and the Institutional Animal Care and Use Committee (IACUC) of Shandong University of Traditional Chinese Medicine.

#### 2.10.2 Histological observations

To evaluate histological changes in the kidney, the animals were euthanized, and their kidneys were removed and quickly immersed in 4% paraformaldehyde for 24 h. The kidney tissue was dehydrated, embedded in paraffin, and sectioned at a thickness of 3 μm. Finally, hematoxylin-eosin staining was performed, and the pathological changes in the animals’ kidney tissue were observed under a light microscope at 400-fold magnification.

#### 2.10.3 Western blot analysis

The kidney tissue was ground in liquid nitrogen and cleaved with a strong RIPA buffer (EA0002, Sparkjade). The protein concentration was determined using a bicinchoninic acid (BCA) protein quantitation kit (EA0002, Sparkjade). Primary antibodies targeting NF-κB p65 (ab19870, Abcam), NF-κB p65 (phospho-S536) (ab76302, Abcam), and beta-actin (20536-I-AP, Proteintech) were incubated overnight with the target protein at 4 °C. The samples were then incubated with HRP-conjugated secondary antibodies (EF0002, Sparkjade). Protein expression was measured using an enhanced chemiluminescence reagent (ECL) kit (ED0015, Sparkjade).

## 3 Results

### 3.1 Botanical drugs for the treatment of HN

Information on 2,055 patients with HN was collected; the information included 1,499 prescriptions, including 425 botanical drugs that were used 35,734 times. The hierarchical network extraction algorithm identified the core botanical drugs, they included Astragalus mongholicus Bunge [Fabaceae; Astragalus mongholicus radix et rhizoma] (huanqi) 15–25 g/d, Salvia miltiorrhiza Bunge [Lamiaceae; Salviae miltiorrhizae radix et rhizoma] (danshen) 10–20 g/d, Codonopsis pilosula (Franch.) Nannf. [Campanulaceae; Codonopsis pilosula (Franch.) Nannf. radix et rhizome] (dangshen) 10–30 g/d, Poria cocos [Polyporaceae; Poria cocos dry sclerotia] (fuling) 15–30 g/d, Atractylodes macrocephala Koidz. [Asteraceae; Atractylodes macrocephala Koidz. radix et rhizome] (baizhu) 10–20 g/d, Angelica sinensis (Oliv.) Diels [Apiaceae; Angelica sinensis(Oliv.)Diels radix et rhizome] (danggui) 15–30 g/d, Citrus × aurantium f. deliciosa Pericarpium [Rutaceae; Citrus × aurantium f. deliciosa dry pericarpium] (chenpi) 10–20 g/d, Pinellia ternata (Thunb.) Makino [Araceae; Pinellia ternata (Thunb.) Makino radix et rhizome] (banxia) 3–6 g/d, and Glycyrrhiza uralensis Fisch. [Fabaceae; Glycyrrhiza uralensis Fisch. radix et rhizome] (gancao) 3–10 g/d. Five traditional Chinese medicines with the highest RR values and *p* < 0.05 were retained as highly specific botanical drugs, they were Dianthus superbus L. [Caryophyllaceae; Dianthus superbus L. radix et rhizome] (qumai) 3–10 g/d, Plantago asiatica L. [Plantaginaceae; Plantago asiatica L. mature seeds] (cheqianzi) 10–20 g/d, Curcuma phaeocaulis Valeton [Zingiberaceae; Curcuma phaeocaulis Valeton radix et rhizome] (ezhu) 10–20 g/d, Euryale ferox Salisb. [Nymphaeaceae; Euryale ferox Salisb. mature seeds] (qianshi) 10–20 g/d, and Achyranthes bidentata Blume [Amaranthaceae; Achyranthes bidentata Blume radix et rhizome] (niuxi) 15–30 g/d. The botanical drugs in the EMR data were boiled for 30 min and filtered. The combination of two parts with a total of 14 botanical drugs produced 961 compounds and 1,328 regulatory targets from public databases.

### 3.2 Symptom community

**FIGURE 2:**
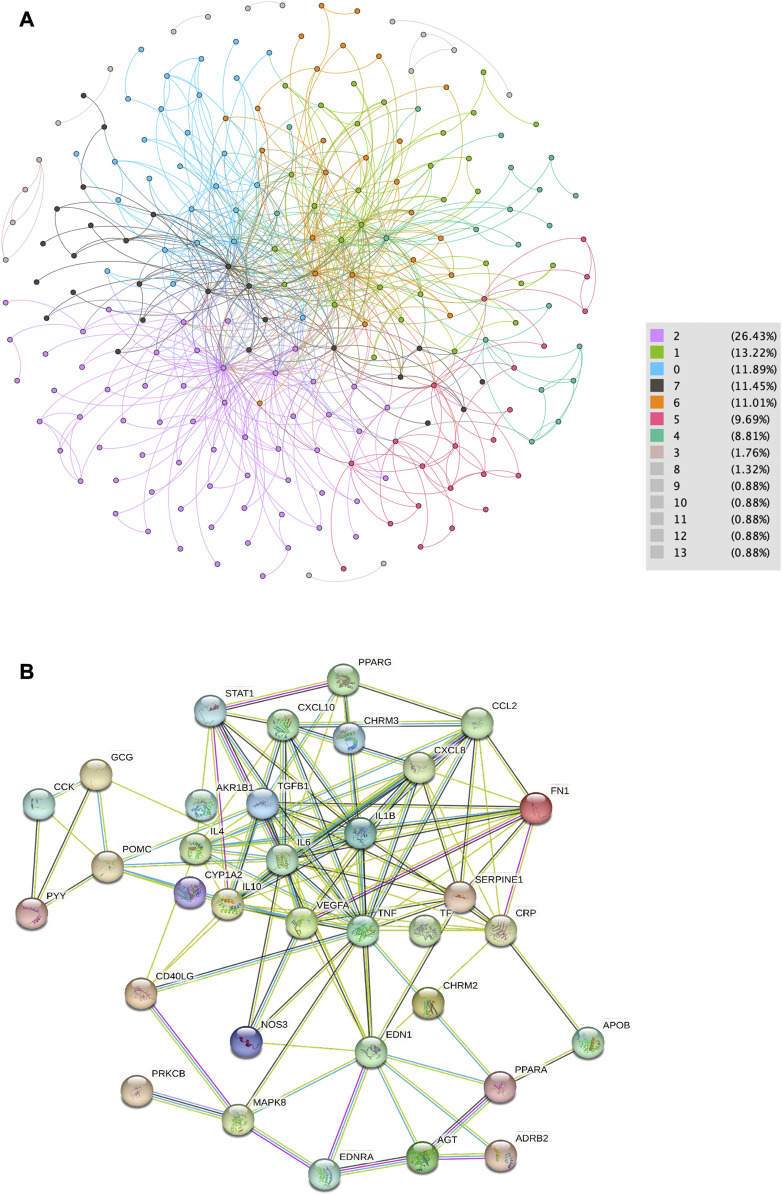
Data collation from EMRs. **(A)** Network map of symptom distribution in patients with HN. **(B)** Core nodes of the PPIN.

A total of 244 symptoms were extracted from EMRs. The fast unfolding algorithm was used to divide the symptoms into 14 network communities, as shown in Figure 2A; M2, M1, M0, M7, and M6 were the main symptom communities. The M2 core symptoms were elevated serum creatinine, urgent urination, and elevated blood pressure; M1 was wheezing, M0 was a loss of appetite, M6 was a cough, and M7 was a headache.

### 3.3 PPIN and core targets

As shown in Figure 2B, 1,017 botanical drug-related compounds, 1,414 regulatory targets, 42 HN-related genes, and M0-, M1-, M2-, M6-, and M7-related genes were collected from the public database, and STING was used in the online analysis to retain high-confidence targets to establish the PPIN. The Dijkstra algorithm was used to identify the core nodes in the PPIN. A total of 61 core targets were retained in 14 botanical drugs, 43 genes related to HN, and 179 genes related to the symptoms.

### 3.4 KNN model

**FIGURE 3:**
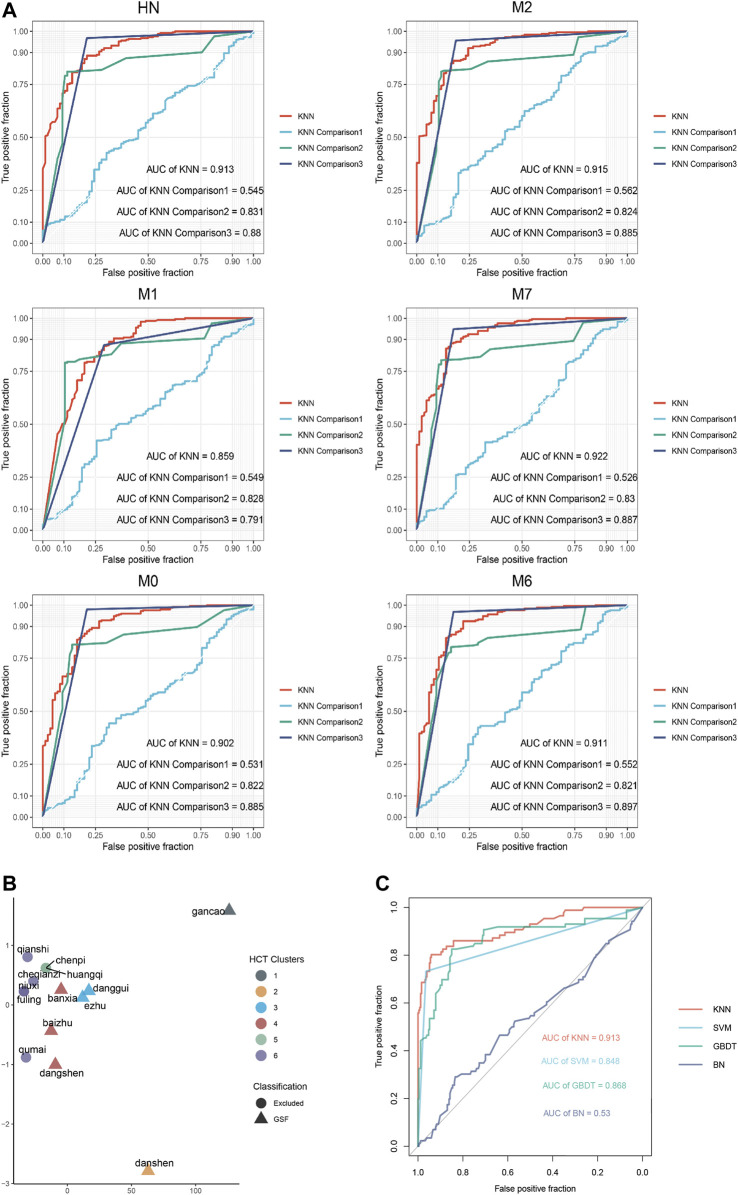
Results obtained using the machine learning model. **(A)** ROC curves obtained using the KNN model. **(B)** Hierarchical clustering of botanical drugs. **(C)** ROC curves obtained using each comparison model.

The biometric information on the quantized compound was taken as the input information. The ROC curve of HN and its symptom community (Figure 3A) shows that the training model based on the overall biometric information of compounds was the best; the AUC of each module was greater than 0.90.

### 3.5 Botanical drug composition of GSF

According to the statistics on the optimal number of compounds in each module of 14 core traditional Chinese medicines, a distance matrix was established, and HCT was used to classify the combinations of botanical drugs (Figure 3B). Salvia miltiorrhiza Bunge, Angelica sinensis (Oliv.) Diels, Atractylodes macrocephala Koidz, Codonopsis pilosula (Franch.) Nannf, Pinellia ternata (Thunb.) Makino, Curcuma phaeocaulis Valeton, and Glycyrrhiza uralensis Fisch. were comprehensively regulated and closely related in a manner that was statistically significant (*p* < 0.05). This combination of botanical drugs was used as an effective prescription, Gao Shen Formula (GSF), for HN.

### 3.6 Model verification

Based on the ROC curves obtained for each model (Figure 3C), the KNN model (AUC = 0.913) was better than the classical models (AUC_SVM_ = 0.845, AUC_GBDT_ = 0.868, and AUC_BN_ = 0.530). This shows that the training setting of the KNN model was reasonable and had a stable prediction performance.

### 3.7 GSF target

**FIGURE 4:**
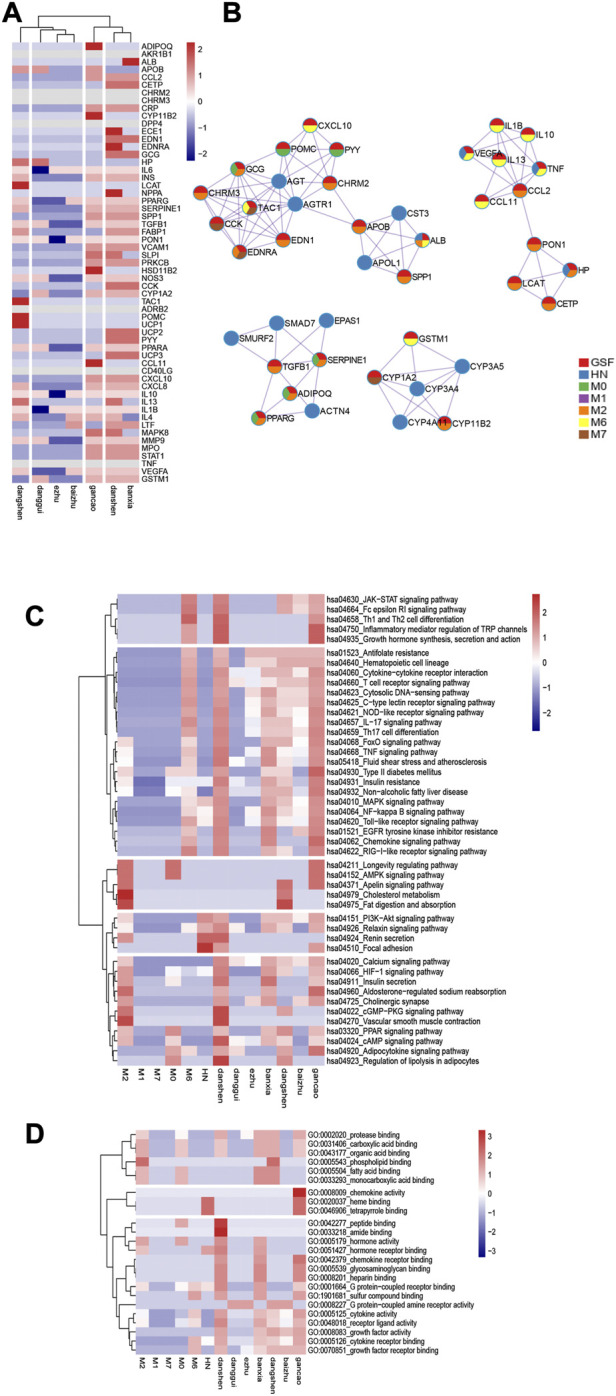
Characteristics of the GSF pathway. **(A)** Target characteristics of GSF. **(B)** GSF and HN interactive network. **(C)** and **(D)** are the KEGG pathway and GO enrichment heatmaps of the GSF and disease modules. In the map, the average number of enriched genes is 0; numbers higher than the average are shown in red, and numbers lower than the average are shown in blue.

GSF contains 57 core targets. To show its biological characteristics, a 7 × 57 matrix was established for the heatmap (Figure 4A). Among the targets, AKR1B1, CHRM2, CHRM3, DPP4, ADRB2, CD40LG, TNF, IL6, PON1, IL10, and IL1B, most of which are related to inflammation, are the most common targets of botanical drugs. Salvia miltiorrhiza Bunge, Codonopsis pilosula (Franch.) Nannf., Pinellia ternata (Thunb.) Makino, and Glycyrrhiza uralensis Fisch. had the most extensive enrichment targets. The MCODE algorithm was used to classify GSF and each symptom module in the PPIN (Figure 4B), the results show that these GSF targets can regulate HN and its symptoms.

### 3.8 GSF pathway

GSF was enriched in 46 KEGG pathways and 24 GO terms. Combined with the enrichment results for each symptom community, 13 × 46 and 13 × 24 matrices were established based on the number of enriched genes. The enrichment pathways are presented as heatmaps (Figures 4C, D). The KEGG pathways and the GO entries were divided into five groups and three groups, respectively.

Among the enriched KEGG pathways, Salvia miltiorrhiza Bunge, Codonopsis pilosula (Franch.) Nannf., Pinellia ternata (Thunb.) Makino, and Glycyrrhiza uralensis Fisch. were significantly enriched in inflammation-related pathways such as the NF-κB signaling pathway, the HIF-1 signaling pathway, the TNF signaling pathway, and the NOD-like receptor signaling pathway and can regulate vascular endothelial proliferation pathways such as the MAPK signaling pathway, the PI3K-Akt signaling pathway, and the VEGF signaling pathway. In addition, Salvia miltiorrhiza Bunge can regulate the cGMP-PKG signaling pathway, act on vascular smooth muscle, and regulate blood pressure. At the same time, Salvia miltiorrhiza Bunge can regulate the renin secretion pathway and the PI3K-Akt signaling pathway, regulate downstream inflammatory factors after renin activation, inhibit renal interstitial fibrosis, and antagonize the renin-angiotensin-aldosterone system. Glycyrrhiza uralensis Fisch. not only inhibits inflammation but also regulates the endocrine system, regulating protein, fatty acid, and glycogen metabolism by acting on the AMPK signaling pathway. Codonopsis pilosula (Franch.) Nannf. can also regulate fat and carbohydrate metabolism by affecting the PPAR signaling pathway. Angelica sinensis (Oliv.) Diels can regulate the cAMP pathway and affect the cytoskeleton. Euryale ferox Salisb. and Atractylodes macrocephala Koidz. can also act on cholinergic synapses and on the calcium signaling pathway, affect smooth muscle, and regulate blood pressure.

Based on the obtained GO terms, GSF mainly regulates cytokines, chemokines, and growth factors. Salvia miltiorrhiza Bunge, Glycyrrhiza uralensis Fisch. and Pinellia ternata (Thunb.) Makino showed the most extensive regulation of cellular processes, including the regulation of DNA transcription factors and the production of a variety of amino acids.

### 3.9 Clinical effect

The treatment plans of a total of 286 patients with HN in the EMRs included GSF, and 279 individuals did not receive TCM treatment. Based on our calculation of sample size, 91 patients were needed in each group. To ensure that the baseline levels of the patients in the groups were consistent, the patients in each group were excluded according to IPTW, (Table 1) and 91 patients were included in the GSF group and 91 patients in the control group (Table 2).

**TABLE 1:** Results before and after propensity score matching of HN cases.

		*p* value	Standardized mean difference
Sex	Before matching	0.335	0.152
	After matching	0.878	0.045
Age	Before matching	0.341	0.131
	After matching	0.949	0.01
Hospital stay duration	Before matching	0.483	0.096
	After matching	0.778	0.056
Glomerular filtration rate	Before matching	0.33	0.134
	After matching	0.704	0.056
Blood urea nitrogen	Before matching	0.027	0.306
	After matching	0.943	0.011
Urine protein	Before matching	0.505	0.113
	After matching	0.744	0.073
CKD stages	Before matching	0.336	0.296
	After matching	0.427	0.294

**TABLE 2:** Characteristics of HN cases.

Demographics and clinical characteristics	GSF group (*n* = 91)	Non-TCM group (*n* = 91)	*p*-value
Age	62.35 ± 17.67	62.52 ± 17.16	0.949
Sex (male)	56(61.5%)	58 (63.7%)	0.878
Hospital stay duration (day)	16.62 ± 7.54	16.92 ± 7.15	0.778
Liver function			
Alanine transaminase (U/L)	20.36 ± 33.55	22.13 ± 28.01	0.7
Aspartate transaminase (U/L)	21.62 ± 15.37	28.04 ± 18.13	0.137
Glucolipid metabolism			
Triglyceride (mmol/L)	2.18 ± 0.78	1.77 ± 0.93	0.145
Cholesterol (mmol/L)	4.42 ± 1.20	4.68 ± 1.81	0.706
Low-density lipoprotein (mmol/L)	2.69 ± 1.27	2.7 ± 1.24	0.961
High-density lipoprotein (mmol/L)	1.1 ± 0.39	1.05 ± 0.41	0.443
Apolipoprotein A1 (g/L)	1.39 ± 0.27	1.36 ± 0.43	0.659
Apolipoprotein B (g/L)	1.08 ± 0.52	1.09 ± 0.52	0.858
Fasting plasma glucose (mmol/L)	5.83 ± 1.81	6.13 ± 2.9	0.408
Kidney function			
Uric acid (μmol/L)	424.03 ± 130.14	419.92 ± 131.01	0.832
Blood urea nitrogen (mmol/L)	13.62 ± 9.03	13.52 ± 9.28	0.943
Urine protein (positive)	63 (69.2%)	66 (72.5%)	0.744
Serum creatinine (μmol/L)	263.65 ± 131.28	248.82 ± 148.35	0.704
Glomerular filtration rate [ml/(min*1.73m^2^)]	44.66 ± 33.34	45.45 ± 33.15	0.873
CKD stages (count)			0.427
Stage 1	14 (15.4%)	14 (15.4%)	
Stage 2	28 (30.8%)	20 (22%)	
Stage 3	23 (25.3%)	32 (35.2%)	
Stage 4	20 (22%)	16 (17.6%)	
Stage 5	6 (6.6%)	9(9.9%)	

Data are presented as mean ± SD, and n (%). *p* values were calculated *via* Student’s t-test or χ2 test as appropriate.

There was no significant difference in age, sex, length of stay, liver function, glucose metabolism, lipid metabolism, or renal function among the enrolled patients (*p* > 0.05). After 6 months of treatment, the creatinine clearance rate in both groups increased (*p* < 0.05), the serum creatinine level in the GSF group was lower than that in the control group (*p* < 0.05), and more patients in the GSF group than in the control group experienced a decrease in the CKD stage [RR = 1.498, 95% CI (1.034–2.168), *p* < 0.05]. Based on this retrospective cohort validation, the addition of GSF to conventional Western medical treatment improves the renal function of patients and reduces their serum creatinine levels, and there are no evident adverse reactions.

### 3.10 *In vivo* experiment

The composition of GSF includes Salvia miltiorrhiza Bunge [Lamiaceae; Salviae miltiorrhizae radix et rhizoma] (15 g), Angelica sinensis (Oliv.) Diels [Apiaceae; Angelica sinensis (Oliv.) Diels radix et rhizome] (9 g), Astragalus mongholicus Bunge [Fabaceae; Astragalus mongholicus radix et rhizoma] (9 g), Codonopsis pilosula (Franch.) Nannf. [Campanulaceae; Codonopsis pilosula (Franch.) Nannf. radix et rhizome] (15 g), Pinellia ternata (Thunb.) Makino [Araceae; Pinellia ternata (Thunb.) Makino radix et rhizome] (9 g), Curcuma phaeocaulis Valeton [Zingiberaceae; Curcuma phaeocaulis Valeton radix et rhizome] (9 g), and Glycyrrhiza uralensis Fisch. [Fabaceae; Glycyrrhiza uralensis Fisch. radix et rhizome] (6 g). The botanical drugs were boiled in water for 1 h, filtered, concentrated, dried, and pulverized to make granules. Granules of botanical drugs were purchased from Tiangjiang Pharmaceutical Co., Ltd, China.

After 8 weeks of drug treatment of SHR rats, systolic blood pressure and diastolic blood pressure decreased in the GSF group and in the irbesartan group (*p* < 0.05) and were lower than those in the model group (*p* < 0.05). There was no difference in blood pressure between the GSF group and the irbesartan group after treatment (*p* > 0.05). It is suggested that both GSF and irbesartan have hypotensive effects on SHR.

**FIGURE 5:**
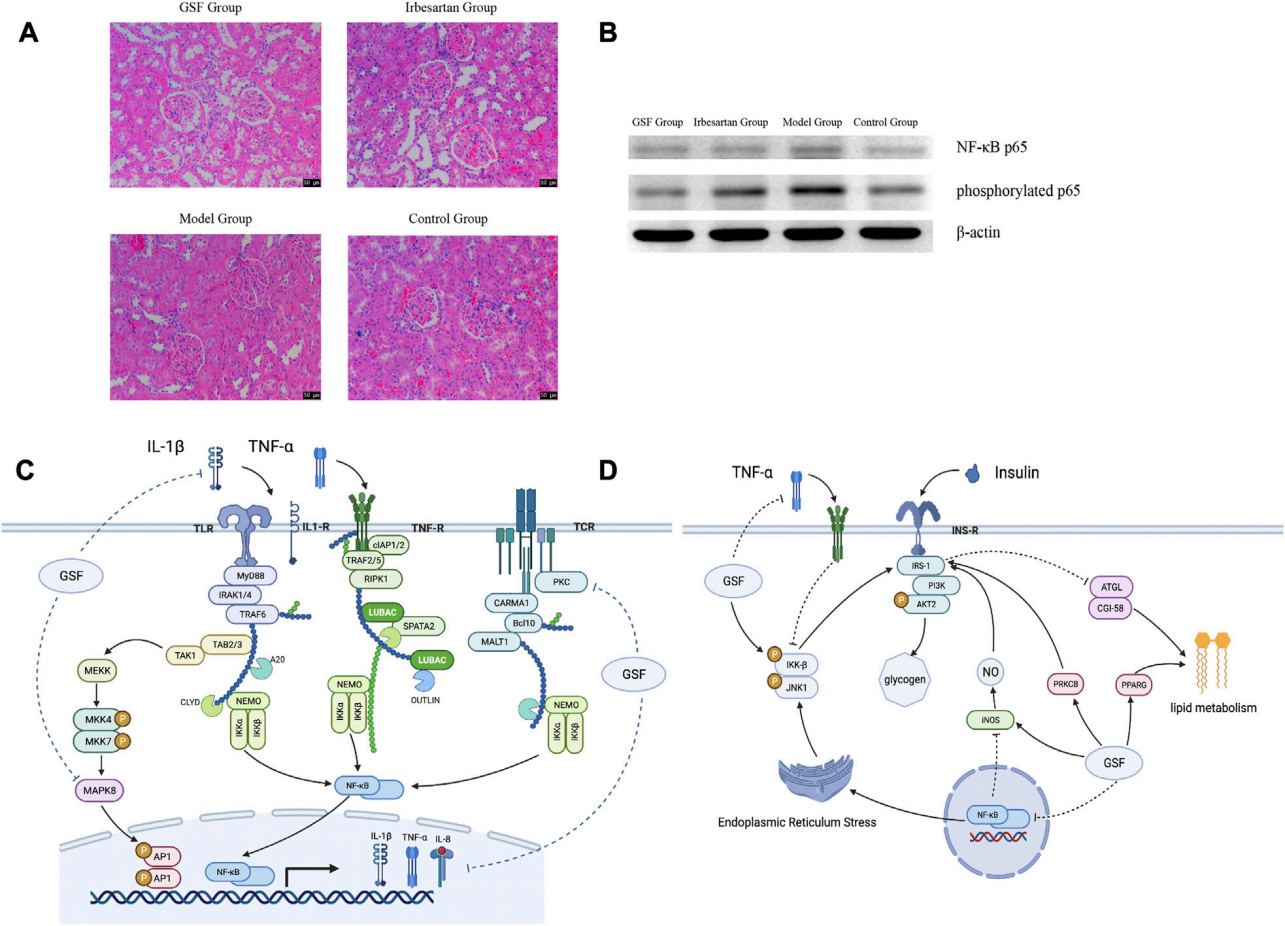
Mechanism of action of GSF. **(A)** HE staining of kidneys at ×200 magnification. **(B)** Expression of NF-κB p65 in the kidney. **(C)** GSF regulates the NF-κB signaling pathway; it can interfere with the production of the IKK complex in the NF-κB pathway and inhibit NF-κB protein phosphorylation by regulating TNF-α, IL-1β, and PKC. At the same time, GSF can also reduce AP1 phosphorylation and reduce the production of inflammatory factors by affecting MAPK8. **(D)** GSF regulates the downstream inflammatory reaction, regulates the NF-κB pathway, reduces the secretion of inflammatory factors, and improves IRS-1 activity, insulin resistance, and lipid metabolism directly or indirectly by inhibiting the IKK-β complex.

The interventricular septum at the end of diastole, the left ventricular posterior wall, and the left ventricular posterior wall at the end of systole were thinner in the GSF group and the irbesartan group than in the model group (*p* < 0.05). The left ventricular ejection fraction of the rats in all four groups was in the normal range, and there was no significant difference among the groups (*p* > 0.05). It is suggested that GSF can antagonize ventricular hypertrophy caused by hypertension and that its effect is similar to that of irbesartan. As shown in Figure 5A, HE staining revealed that the glomerular structure of the GSF group was clear and complete, without red blood cell aggregation, and there were a few inflammatory cells in the glomeruli. In the irbesartan group, the glomerular capillaries were dilated with mild congestion, and there were a few inflammatory cells in the renal interstitium. In the model group, glomerular mesangial hyperplasia, inflammatory cells in the glomeruli, necrosis and abscission of renal tubular epithelial cells, glomerular mesangial hyperplasia, infiltration of the glomeruli by inflammatory cells, inflammatory cells in the renal interstitium, and thickening of the renal capsule wall were observed. The structure of the glomeruli and tubules in the control group was normal. The serum TNF-α level of the model group was higher than that of the other groups (*p* < 0.05), and there were no significant differences in the serum TNF-α levels of the high kidney formula group, the irbesartan group and the control group (*p* > 0.05). As shown in Figure 5B, the expression of NF-κB p65 and phosphorylated p65 in the SHR kidney was higher in the model group than in the other groups. NF-κB p65 expression in the GSF group was similar to that in the irbesartan group, and phosphorylated p65 showed the lowest expression in the GSF group.

## 4 Discussion

GSF mainly targets inflammation-related pathways and their downstream pathways. Among inflammatory pathways, GSF can not only inhibit the NF-κB pathway and the MAPK signaling pathway, which are activated simultaneously, but can also inhibit TNF-α, IL-1β, and other inflammatory factors and thereby reduce NF-κB transcription (Figure 5C). In its downstream pathway, GSF can not only inhibit insulin resistance induced by inflammatory cytokines but can also regulate glucose and lipid metabolism by directly or indirectly increasing IRS-1 activity (Figure 5D).

Arteriosclerosis and hyaline degeneration caused by hypertension are the main pathological changes that are closely related to inflammation and renal tubulointerstitial fibrosis (Seccia et al., 2017). Under physiological conditions, the internal pressure within the glomerulus is relatively constant. When the blood pressure increases, the small entering arteries contract appropriately and reduce the pulse pressure difference, protecting the glomerulus. When the self-regulation of renal microcirculation weakens, the small artery entering the glomerulus expands abnormally, and the internal pressure in the glomerulus increases. At the same time, the hypertension of the large artery and the high pulse pressure difference are transmitted to the glomerulus, causing internal pulsation and stretching and resulting in endothelial damage (Stompor and Perkowska-Ptasińska, 2020). These hemodynamic changes simultaneously activate the RAAS system, aggravate the inflammatory reaction, and cause interstitial fiber hyperplasia, arteriolar thickening, and oxidative stress reaction of the kidney tissue, thereby damaging the function of the nephron.

After excessive activation of the RAAS, the secretion of angiotensin II (Ang II) increases, causing upregulation of the production of inflammatory cytokines such as TNF-α, IL-6, and IL-8 expression in kidney tissue. The mRNA and protein levels of type I and IV collagen are increased (Henke et al., 2007; Lu et al., 2019; Mocker et al., 2019), and excessive expression of Ang II receptors on podocytes promotes the disappearance of foot processes (Hoffmann et al., 2004), induces reorganization of the podocyte cytoskeleton, and causes proteinuria (Li et al., 2019). In renal tubular epithelial cells, Ang II stimulates fibroblasts to form an extracellular matrix at the injured site, resulting in fibrosis and renal tubular epithelial mesenchymal transformation (He et al., 2018; Said et al., 2018; Cavalcante et al., 2019; Seccia et al., 2019).

According to the pathway enrichment results, the inflammatory pathway related to hypertensive renal damage that is regulated by GSF is mainly the NF-κB signaling pathway. GSF may regulate TNF-α, IL-1β, IL-6, and other inflammatory factors indirectly or directly, thereby inhibiting the NF-κB signaling pathway and further inhibiting the inflammatory response caused by inflammatory cytokines, the upregulation of the MAPK signaling pathway caused by MAPK8 and TNF, and the interstitial fibrosis caused by the increase in serine (Meng et al., 2016; Ni et al., 2020). GSF treatment can also improve the M6 respiratory tract inflammation community and the M7 pain community, both of which are also rich in inflammatory pathways.

In the early stage of hypertensive renal damage, glomerular metabolism increases, especially GTP metabolism, lipid breakdown, and the production of various amino acids. Moreover, due to the increase in lipid oxidation, an inflammatory reaction can be induced (Dennis and Norris, 2015), and this can trigger changes in the cytoskeleton, leading to foot cell damage (Rinschen et al., 2019).

The drugs contained in GSF can regulate insulin resistance and the PPAR signaling pathway; improve the utilization of lipids and sugars by the body through specific effects on INS, PPARG, ADIPOQ, and other targets (especially fat metabolism regulated by insulin); and improve the efficiency of utilization of sugars and lipids (Feige et al., 2006; Frühbeck et al., 2014). GSF can also regulate and inhibit changes in the cytoskeleton, reduce the migration of glial cells, improve vascular endothelial function (Bance et al., 2019; Natale et al., 2019), inhibit processes that damage podocytes, protect nephrons, and improve the symptoms of kidney damage in the M2 community by interfering with VEGFA, PRKCB, and JNK in the focal adhesion pathway.

To verify the anti-inflammatory mechanism of GSF, this study used SHR rats to establish a disease model. After eight weeks of intervention with GSF, it was determined that GSF reduced renal NF-κB p65 and phosphorylated p65 levels, indicating that NF-κB dimer decreased, signal conduction was weakened, and NF-κB activation was inhibited, thereby reducing the production of inflammatory factors similar to serum TNF-α. Fewer inflammatory cells were present in the pathological sections obtained from the kidneys of the animals in the GSF group, and structural changes in the nephrons were not notable, indicating that GSF alleviates renal inflammation. The animals in the GSF group also had decreased blood pressure after intervention. The Color Doppler ultrasound showed that the left ventricular myocardium was thinner in the animals in the GSF group than in those in the model group, proving that it delays myocardial remodeling. No adverse reactions or death were observed during the experiment, which could preliminarily indicate its safety.

This research innovatively combines network pharmacology with real-world data mining and uses the KNN model to process information on diseases and botanical drugs, closely linking systems biology with clinical data. To determine the efficacy of the model, it was compared with other classical machine learning models, and its efficacy was verified in a retrospective cohort. Finally, animal experiments were used to verify the core mechanism of action of GSF. The results show that compared with traditional models, the KNN model can better handle the heterogeneity and complexity of the relationship between genome data and TCM clinical information. GSF obtained using this method was shown to have a practical effect after verification, and the findings have a certain reference significance for the analysis of target organ damage after TCM treatment of hypertension.

At the same time, this study also has limitations. First, although the symptoms of the patients are included in the evaluation system, this study still uses the patients as a whole dataset and fails to make full use of the complex clinical individualized differences reflected by the real-world data. Second, the sample size used for model training in this study was limited. Although we used a variety of models for comparative verification, further research is needed to improve the model’s performance. Third, the animal experiments conducted in this study only verified the most fundamental anti-inflammatory mechanism, and no experimental analysis of the changes in downstream pathways of the inflammatory response was performed; thus, further research on the pathways and mechanisms involved is needed. In terms of clinical effects, this study features a small sample size, multiple risks of bias, heterogeneity of GSF among patients, and limited measurement of results. In addition, randomized trials should be conducted to determine the efficacy and safety of GSF in the treatment of HN (Tables 1, 2).

## 5 Conclusion

This study was based on EMR data for patients with HN collected at the Affiliated Hospital of Shandong University of Traditional Chinese Medicine from 2014 to 2017. It identified core botanical drugs for the treatment of HN. After establishment of the PPIN, machine learning model training was used to obtain an effective compound screening model based on information on the biological characteristics of compounds, and a hierarchical clustering screening of botanical drugs was conducted based on the model results, retaining the important botanical drugs as GSF for treating HN. From the perspective of targets and pathways, it was found that GSF plays a multilevel biological regulatory role in which it controls inflammatory reactions that lead to hypertensive kidney damage and reduces the damage caused by excessive activation of the RAAS system.

## Data Availability

The original contributions presented in the study are included in the article/Supplementary Material; further inquiries can be directed to the corresponding authors.

## Glossary

Achyranthis Bidentatae: niuxi
Angelicae Sinensis Radix: danggui
Angiotensin II: Ang II
Astragali Radix: huanqi
Atractylodis Macrocephalae Rhizoma: baizhu
Bayesian network: BN
Chronic kidney disease: CKD
Citri Reticulatae Pericarpium: chenpi
Codonopsis Radix: dangshen
Curcumae Rhizoma: ezhu
Dianthi Herba: qumai
Electronic medical record: EMR
Euryales Semen: qianshi
Gao Shen Formula: GSF
Gene Ontology: GO
Glycyrrhizae Radix Et Rhizoma: gancao
GO biological process semantic similarity: GoSim
Gradient boosting decision tree: GBDT
Hierarchical clustering algorithm: HCT
Hypertensive nephropathy: HN
K-nearest neighbor: KNN
Kyoto Encyclopedia of Genes and Genomes: KEGG
Medical Subject Headings: MeSH
Online Mendelian Inheritance in Man: OMIM
Oral bioavailability: OB
Pinelliae Rhizoma: banxia
Plantaginis Semen: cheqianzi
Poria: fuling
Protein–protein interaction network: PPIN
Real-world data: RWD
Relative risk: RR
Renin-angiotensin-aldosterone system: RAAS
Restarting the random walk: RWR
Salviae Miltiorrhizae Radix Et Rhizoma: danshen
Support vector machines: SVM
Area under curve: (AUC)
The Encyclopedia of Traditional Chinese Medicine: ETCM
Inverse propensity of treatment weighting: IPTW
Jaccard similarity coefficient: Jac
Manhattan distance: Man
Molecular complex detection: MCODE
Receiver operating characteristic: ROC
Traditional Chinese Medicine Systems Pharmacology Database and Analysis Platform: TCMSP
Traditional Chinese medicine: TCM.
